# BDNF-induced LTP is associated with rapid Arc/Arg3.1-dependent enhancement in adult hippocampal neurogenesis

**DOI:** 10.1038/srep21222

**Published:** 2016-02-18

**Authors:** Sjoukje D. Kuipers, Andrea Trentani, Adrian Tiron, Xiaosong Mao, Dietmar Kuhl, Clive R. Bramham

**Affiliations:** 1 Department of Biomedicine and KG Jebsen Centre for Research on Neuropsychiatric Disorders, Jonas Lies vei 91, University of Bergen, 5009 Bergen, Norway; 2 Institute for Molecular and Cellular Cognition, Center for Molecular Neurobiology (ZMNH) University Medical Center Hamburg-Eppendorf, Falkenried 94, 20251 Hamburg, Germany

## Abstract

Adult neurogenesis in the hippocampus is a remarkable phenomenon involved in various aspects of learning and memory as well as disease pathophysiology. Brain-derived neurotrophic factor (BDNF) represents a major player in the regulation of this unique form of neuroplasticity, yet the mechanisms underlying its pro-neurogenic actions remain unclear. Here, we examined the effects associated with brief (25 min), unilateral infusion of BDNF in the rat dentate gyrus. Acute BDNF infusion induced long-term potentiation (LTP) of medial perforant path-evoked synaptic transmission and, concomitantly, enhanced hippocampal neurogenesis bilaterally, reflected by increased dentate gyrus BrdU + cell numbers. Importantly, inhibition of activity-regulated cytoskeleton-associated protein (Arc/Arg3.1) translation through local, unilateral infusion of anti-sense oligodeoxynucleotides (ArcAS) prior to BDNF infusion blocked both BDNF-LTP induction and the associated pro-neurogenic effects. Notably, basal rates of proliferation and newborn cell survival were unaltered in homozygous Arc/Arg3.1 knockout mice. Taken together these findings link the pro-neurogenic effects of acute BDNF infusion to induction of Arc/Arg3.1-dependent LTP in the adult rodent dentate gyrus.

BDNF plays a central role in neuronal maturation, circuit formation, and activity-dependent forms of plasticity such as LTP of synaptic transmission. In the adult brain, endogenous BDNF instructs gene expression and protein synthesis underlying LTP and memory formation, while exogenous application of BDNF has been found to induce LTP (so-called BDNF-LTP) in many forebrain excitatory synapses^1^,^2^,^3^,^4^. However, BDNF-mediated actions in the adult brain are not limited to synaptic plasticity but extend to neurogenesis, the birth of new functional neurons from adult neural progenitor cells (NPCs). In the dentate gyrus (DG), new neurons are continuously generated throughout life, endowing this structure with a remarkable structural plasticity essential for its roles in learning and memory formation^5^,^6^. BDNF has been shown to stimulate proliferation of NPCs and promote long-term survival of their progeny^7^,^8^,^9^,^10^,^11^. Both intra-hippocampal infusion of BDNF and its peripheral injection have been associated with potent stimulation of hippocampal neurogenesis^9^,^12^,^13^.

The mechanisms by which BDNF administration promotes NPC proliferation in the DG have not been established. Exogenous BDNF may act directly on the progenitor population or through indirect mechanisms. Previously, LTP induced by high-frequency stimulation of perforant path input to dentate granule cells has been shown to lead to enhanced neurogenesis^14^,^15^, raising the possibility that BDNF-induced LTP similarly triggers enhanced proliferation. However, to date no studies have examined neurogenesis under conditions of acute BDNF infusion that induce LTP.

Expression of the immediate early gene Arc/Arg3.1 is required for development of both BDNF-LTP and high frequency stimulation (HFS)-LTP at medial perforant path-granule cell synapses in the DG^16^,^17^,^18^. Following stimulation of the performant path, Arc/Arg3.1 mRNA is induced in adult dentate granule cells and undergoes rapid transport to dendrites and local translation^19^,^20^,^21^,^22^. During LTP consolidation, dynamic synthesis of Arc/Arg3.1 is required for stable expansion of the actin cytoskeleton at perforant path synapses. Inhibition of Arc/Arg3.1 synthesis with anti-sense oligodeoxynucleotides therefore blocks development of stable LTP^17^.

Here, we asked whether BDNF-LTP is associated with enhanced hippocampal NPC proliferation. BDNF was locally and unilaterally infused in the DG of adult male rats (Fig. 1). Immediately prior to BDNF, Arc/Arg3.1 anti-sense oligodeoxynucleotides (ODNs) (“ArcAS”) or scrambled Arc ODN sequence control (“ArcASScr”) were also administered locally and unilaterally. We hypothesized that if BDNF-mediated pro-neurogenic actions were mediated by LTP induction, inhibition of Arc/Arg3.1 translation would block these effects. New evidence supports the involvement of Arc/Arg3.1 in the regulation of hippocampal neurogenesis^10^,^23^. While Arc/Arg3.1 induction in ‘older’ adult-born dentate granule cells with mature functional synapses is required for LTP consolidation, Arc/Arg3.1 expression in newborn cells (prior to synapse development) strongly correlates with long-term survival and differentiation towards a neuronal phenotype^10^.

We report that acute unilateral infusion of BDNF is associated with a bilateral increase in BrdU+ cell numbers in the DG. Moreover, unilateral infusion of ArcAS blocked bilateral changes in proliferation, indicating that the BDNF-induced proliferative response is a consequence of the unilateral induction of Arc/Arg3.1-dependent BDNF-LTP. We also compared basal levels of proliferation between Arc/Arg3.1 knockout and wild-type mice and found no significant differences. Together these data suggest that exogenous BDNF enhances neurogenesis indirectly by a mechanism involving LTP induction in the adult granule cell population. The data do not rule out a role for Arc/Arg3.1 synthesis in progenitor cells in neurogenesis.

## Results

Previous studies have shown that brief infusion of BDNF into the dentate gyrus induces LTP. In the present study, BDNF was infused immediately above the DG, 300μm dorsal to the synaptic zone of medial perforant path fibers. Previous immunohistochemical analysis of BDNF distribution showed rapid delivery of BDNF to the dorsal dentate gyrus (within <15 min of infusion onset) and clearance from the dentate gyrus in less than 1 hour; BDNF also diffuses along the cannula tract in the CA1 region, with variable spread to CA3^24^. BDNF infusion promotes the induction of LTP which lasts for at least 15 hours in anesthetized rats, rapidly activates the extracellular signal regulated protein kinase (ERK)^25^,^26^ and requires transcription of new Arc mRNA and new Arc protein synthesis^17^,^24^. Accordingly, in the present experiment, BDNF infusion led to a robust increase in the fEPSP slope and population spike amplitude, an effect which was inhibited by ArcAS infusion but not ArcASScr (Fig. 2). Statistical analysis (general linear model for repeated measures) indicated a significant main effect of time (baseline vs infusion vs recording: F = 425.801 (Wilks’ Lambda), p < 0.0001) and a significant interaction between time and treatment (Cytochrome C (CytC) vs BDNF vs BDNF+ArcAS vs BDNF+ArcASScr: F = 31.391, p < 0.0001). Subsequent pair-wise comparisons (Sidak’s post-hoc) indicated a significant difference between animals receiving BDNF and CytC (a protein similar to BDNF in molecular weight and charge that was used to control for possible nonspecific effects of protein infusion; p < 0.001). The effect of BDNF infusion on LTP was completely inhibited by preceding ArcAS administration (BDNF vs BDNF+ArcAS, p < 0.001) but not ArcASScr (BDNF vs BDNF+ArcASScr, p > 0.05; CytC vs BDNF+ArcASSc, p < 0.001). No differences were detected between CytC and BDNF+ArcAS-treated animals (Fig. 2). These results show that infusion of exogenous BDNF in the DG was associated with the induction of robust LTP which requires the synthesis of new Arc protein. Blockage of Arc translation, through the administration of Arc AS (but not Arc ASScrambled) inhibited LTP induction.

### Total labeled (IdU+ and BrdU+) cells

**Figure 1:**
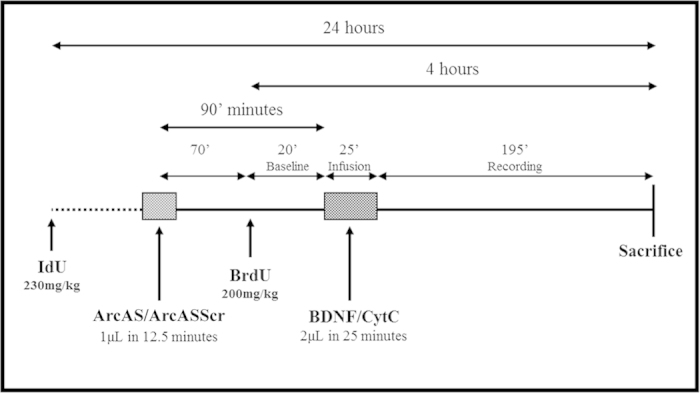
Schematic representation illustrating the sequence of events comprising the experimental setup. IdU (230 mg/kg; i.p.) and BrdU (200 mg/kg; i.p.) were administered 24 and 4 hours, respectively, prior to sacrifice to time-stamp hippocampal newborn cells that underwent division. After a 20 min baseline, BDNF (or Cytochrome C) was infused, 20 minutes following BrdU administration, for 25 minutes at a speed of 80 nl/min. Subsequent BDNF-induced LTP was monitored and recorded for 195 minutes, before animals were sacrificed and tissue was collected for histological analysis. Arc/Arg3.1 antisense oligodeoxynucleotides (Arc AS) or Arc/Arg3.1 scrambled oligodeoxynucleotides (Arc ASScr) were administered 90 minutes prior to BDNF infusion for 12.5 minutes at a rate of 80 nl/min to inhibit Arc/Arg3.1 translation and block BDNF-induced LTP.

Iodo-deoxy-Uridine (IdU) and Bromo-deoxy-Uridine (BrdU) are two thymidine analogs that integrate into cells undergoing DNA synthesis. Their incorporation into the DNA can be visualized through immunohistochemistry to provide an *in vivo* marker to identify proliferating cells. IdU was given to adult male rats 24 hours prior to sacrifice and 20 hours prior to BrdU injection to determine basal proliferative rates prior to BDNF administration. BrdU was administered 4 hours prior to sacrifice to explore BDNF-induced effects on hippocampal NPC proliferation (Fig. 1). Importantly, the anti-IdU antibody does not discriminate between IdU and BrdU, recognizing both analogs and labeling cells which have incorporated IdU and/or BrdU. In contrast, the anti-BrdU antibody selectively detects BrdU without cross-reacting with IdU. As a result of primary antibody specificity, total number of labeled cells thus represents a combination of three distinct cell populations: IdU(only)+ cells, BrdU(only)+ and double IdU+/BrdU+ cells. Total labeled cells (IdU(only)+ and BrdU+) were quantified by light microscopy across the entire rostral-caudal extent of the hippocampus using serial sets of coronal sections (Fig. 3a,b). Two-way ANOVA was run to determine inter-hemispheric differences (BDNF infusion vs control side), treatment effects (CytC vs BDNF vs BDNF+ArcAS) and their interactions (treatment × infusion side). Interestingly, although BDNF was infused in only one hemisphere, no differences in total labeled cell numbers were found between stimulated and non-stimulated sides in any hippocampal region examined (hilus, SGZ and GCL) (Fig. 3d,e). A significant main effect of treatment was found in the SGZ (F_2,44_ = 8.454, p < 0.001), with BDNF infusion markedly increasing total labeled cell numbers (t = 3.651, p < 0.001) while BDNF+ArcAS administration prevented this BDNF-mediated proneurogenic effect (t = 3.500, p = 0.0011) (Fig. 3c). No effects were observed in the hilus or GCL.

Total numbers of labeled cells in the different hippocampal regions were subsequently used in combination with confocal imaging (Fig. 4) to calculate basal proliferative rates (total IdU(only)+, total IdU+/BrdU−/Arc− and total IdU+/BrdU−/Arc+ cells) as well as treatment-induced changes on hippocampal proliferation (total BrdU+, total BrdU+/Arc− and total BrdU+/Arc+ cells) for each individual rat.

### Total BrdU+ cells (IdU-/BrdU+ and IdU+/BrdU+ cells)

As BrdU was given 20 minutes prior to BDNF infusion, total numbers of BrdU+ cells provided a direct measure of BDNF-mediated effects on hippocampal NPC proliferation. Our data showed a significant main effect of treatment in the SGZ (F_2,44_ = 16.391, p < 0.001) but not in the hilus or GCL (Fig. 5a). Interestingly, no main effects were found between the infusion and the contralateral side (Fig. 5b,c). Post-hoc analysis revealed a significant increase of total BrdU+ cells in the SGZ (BDNF vs CytC, t = 5.369, p < 0.001) while concurrent ArcAS administration prevented this effect (BDNF+ArcAS vs BDNF, t = 4.467, p < 0.001) (Fig. 5a). These results suggest that the increased bilateral numbers of BrdU+ cells in response to unilateral BDNF infusion depended on BDNF-LTP induction. This was confirmed by the observation that unilateral infusion of ArcAS blocked BDNF-induced pro-neurogenic actions, bilaterally.

### Total IdU(only)+ cells

Since IdU was given approximately 20 hours prior to BrdU administration and BDNF infusion, quantification of total IdU(only)+ cells (IdU+/BrdU−) provided an indication of basal proliferative rates as these cells divided only in response to endogenous stimuli. IdU+ cells which re-divided in response to BDNF also incorporated BrdU becoming double-positive (IdU+ and BrdU+) and, therefore, excluded from the IdU(only)+ group. Results showed a significant main effect of treatment in the hilus (F_2,44_ = 4.723, p = 0.014), SGZ (F_2,44_ = 4.611, p = 0.015) and GCL (F_2,44_ = 5.066, p = 0.010) (Fig. 5d). No main inter-hemispheric effect was detected (Fig. 5e,f). In addition, a significant interaction was found between the treatment and infusion side in the hilus (F_2,44_ = 5.504, p = 0.007). Post-hoc analysis indicated that BDNF infusion significantly reduced total IdU(only)+ cells in the hilus (CytC vs BDNF, t = 3.009, p = 0.0043), SGZ (t = 3.007, p = 0.0044) and GCL (t = 2.944, p = 0.0052) (Fig. 5d). In the GCL only, concurrent ArcAS administration attenuated BDNF-mediated influences (BDNF+ArcAS vs BDNF, t = 2.553, p = 0.014). In the hilus, BDNF-mediated effects on total IdU(only)+ cells were mostly evident in the stimulated side (CytC vs BDNF, t = 4.467, p = 0.000) (Fig. 5e). These results indicate that BDNF stimulates the re-proliferation of NPCs which divided less than 20 hours before incorporating IdU (IdU+/BrdU− cells). As a consequence of this new BDNF-induced division, these cells incorporated BrdU becoming double-positive (IdU+ and BrdU+) and resulting in a net reduction of the IdU+/BrdU− cell population.

### Total BrdU+/Arc− and BrdU+/Arc+ cells

To evaluate specific influences of BDNF and/or ArcAS, BrdU-labeled cells were also further subdivided into Arc-positive and Arc-negative cells. Under basal conditions, only a small percentage (less than 5%) of BrdU+ cells in the DG is also positive for Arc. A significant main effect of treatment was found on **BrdU**+**/Arc**+ **cells** in the hilus (F_2,44_ = 32.518, p < 0.001), SGZ (F_2,44_ = 59.641, p < 0.001) and GCL (F_2,44_ = 49.924, p < 0.001) (Fig. 6a). A significant main effect of stimulation side was also detected in the hilus (F_1,44_ = 12.977, p < 0.001) (Fig. 6b,c) although no interactions were found in any of the regions. Post-hoc analysis revealed that BDNF infusion increased BrdU+/Arc+ cell numbers (BDNF vs. CytC: hilus, t = 7.099, p = 0.000; SGZ, t = 10.289, p = 0.000; GCL, t = 9.591, p = 0.000) while concurrent ArcAS administration prevented this effect (BDNF vs. BDNF+ArcAS: hilus, t = 6.934, p = 0.000; SGZ, t = 8.435, p = 0.000; GCL, t = 7.338, p = 0.000) (Fig. 6a). In the hilus, greater numbers of BrdU+/Arc+ cells were found in the non-stimulated compared to the stimulated side in response to both BDNF (t = 2.322, p = 0.025) and BDNF+ArcAS (t = 2.213, p = 0.032) (Fig. 6b,c).

With regard to **total BrdU**+**/Arc− cells**, a main effect of treatment was again found in the hilus (F_2,44_ = 10.810, p < 0.001) (Fig. 6d). No main effects of the stimulation side (Fig. 6e,f) or interactions were however detected. Post-hoc analysis revealed a significant reduction of hilar BrdU+/Arc− cells in response to BDNF+ArcAS infusion (CytC vs BDNF+ArcAS; t = 4.439, p = 0.000: BDNF vs BDNF+ArcAS; t = 3.470, p = 0.0012) while BDNF had no effects (Fig. 6d). These findings indicate that unilateral BDNF infusion was associated with a bilateral increase in BrdU+/Arc+ cells (possibly caused by BDNF-LTP), an effect which was blocked by unilateral ArcAS infusion. In contrast, both BDNF and BDNF+ArcAS infusion had no effects on the number of BrdU+/Arc− cell population.

### Total IdU+/BrdU-/Arc- and IdU+/BrdU−/Arc+ cells

IdU+/BrdU− cells were also further subdivided into Arc-positive and Arc-negative cells. With regard to total **IdU**+**/BrdU−/Arc**+ **cell** numbers, a significant main effect of treatment was only seen in the hilus (F_2,44_ = 13.870, p < 0.001) (Fig. 7a). No main effects were detected between hemispheres (Fig. 7b,c) while an interaction between treatment and side of infusion was only found in the hilus (F_2,44_ = 6.683, p = 0.003). Post-hoc analysis revealed significantly increased numbers of IdU+/BDNF−/Arc+ cells in the hilus in response to BDNF infusion (BDNF vs CytC, t = 4.077, p = 0.000) while concurrent BDNF+ArcAS administration prevented this effect (BDNF vs BDNF+ArcAS, t = 4.957, p = 0.000) (Fig. 7a).

With regard to **IdU**+**/BrdU−/Arc− cells**, a main effect of treatment was found in the hilus (F_2,44_ = 4.839, p = 0.013), SGZ (F_2,44_ = 18.161, p < 0.001) and GCL (F_2,44_ = 7.461, p = 0.002) (Fig. 7d). No infusion effect was found between hemispheres in any region (Fig. 7e,f) but a significant interaction between treatment and infusion side was detected in the hilus (F_2,44_ = 10.690, p < 0.001). Post-hoc analysis revealed that while BDNF significantly reduced total IdU+/BrdU−/Arc− cell numbers in the hilus (CytC vs BDNF, t = 2.850, p = 0.007), SGZ (t = 5.858, p < 0.001) and GCL (t = 3.520, p = 0.001), concurrent ArcAS administration prevented these effects (BDNF+ArcAS vs BDNF: hilus, t = 2.536, p = 0.015; SGZ, t = 4.229, p < 0.001; GCL, t = 3.176, p = 0.003) (Fig. 7d). In the hilus, a significant reduction of IdU+/BrdU−/Arc− cells was seen in response to BDNF infusion in the stimulated (CytC vs BDNF, t = 5.043, p = 0.000) (Fig. 7e) but not the contralateral side. This effect was prevented by concurrent ArcAS infusion (BDNF+ArcAS vs BDNF, t = 3.995, p = 0.000). These results are similar to BrdU+cells with the important difference that unilateral BDNF infusion was associated with a bilateral reduction in IdU+/BrdU−/Arc− cells and, again, this effect was blocked by unilateral ArcAS infusion. Remarkably, both BDNF and BDNF+ArcAS infusion had no effects on the number of IdU+/BrdU−/Arc+ cells (except in the hilus).

### Total proliferating and surviving BrdU-labeled cells in Arc/Arg3.1-deficient mice

In view of the importance of Arc synthesis in BDNF-LTP, we investigated levels of basal proliferation and total neurogenesis in Arc/Arg3.1-deficient mice. BrdU was injected in Arc/Arg3.1-deficient mice and wild-type controls 24 hours or 28 days prior to sacrifice to investigate effects associated with reduced Arc/Arg3.1 expression on proliferating and differentiated newborn hippocampal cells (Fig. 8a–d). We hypothesized that if Arc transcription was critical in hippocampal neurogenesis, Arc/Arg3.1-deficient mice would display a significant reduction in the rate of proliferation and/or newborn cell survival compared to wild-type mice with normal Arc expression. If Arc involvement in hippocampal neurogenesis was limited to its role in the generation and maintenance of LTP, its effects on proliferation and/or survival would be limited as these mice were not stimulated (neither chemically with BDNF infusion nor behaviorally). Our data illustrate a significant reduction in the number of BrdU-labeled cells between proliferation and survival experiments in the hilus (F_1,20_ = 17.96, p > 0.001), SGZ (F_1,20_ = 140.06, p > 0.001) and GCL (F_1,20_ = 139.70, p > 0.001), due to the well-documented loss of newborn cells during the first month after cell division (Fig. 8e). Interestingly, no main effects of strain (wild-type vs Arc/Arg3.1-deficient mice) or interactions were found in any hippocampal region examined, indicating that Arc-mediated influences on hippocampal neurogenesis were indirect and linked to its role in the generation and maintenance of LTP.

## Discussion

The present results suggest LTP induction is a central mechanism through which acute BDNF infusion modulates hippocampal neurogenesis. Previous work has implicated enhanced BDNF signaling in the behavioral and cellular efficacy of antidepressant drugs^11^,^12^,^13^,^27^,^28^,^29^,^30^. Notably, a single bilateral infusion of BDNF in the dentate gyrus has been found to produce an antidepressant-like effect in behavioral models of depression^13^. Here, we show that acute unilateral BDNF infusion induces stable LTP in the perforant pathway and enhanced hippocampal cell proliferation exhibited by increased BrdU+ cell numbers. Importantly, although only infused unilaterally, BDNF led to enhanced proliferation, bilaterally. Inhibition of Arc/Arg3.1 translation, through local and unilateral Arc/Arg3.1 anti-sense oligodeoxynucleotide (ArcAS) infusion, blocked both BDNF-LTP induction and bilateral enhancement of hippocampal cell proliferation. This data provides the first evidence that the pro-neurogenic actions of acute BDNF administration are coupled to induction of LTP, illustrating a possible mechanism through which the effects of BDNF are translated, at a systems level, into beneficial behavioral actions.

LTP at medial perforant path-granule cell synapses in the DG requires a period of sustained BDNF-TrkB signaling and Arc/Arg3.1 synthesis^1^,^2^,^17^. During this period TrkB signaling drives Arc/Arg3.1 expression, including its local translation within or near dendritic spines of perforant path synapses^2^. Intra-hippocampal BDNF infusion similarly induces Arc/Arg3.1-dependent LTP. Exogenous BDNF induces Arc/Arg3.1 transcription in adult dentate granule cells and transport of Arc/Arg3.1 mRNA to dendrites^24^,^26^. Both the induction of BDNF-LTP and its time-dependent consolidation are inhibited by treatment with ArcAS^17^,^24^,^26^,^31^,^32^.

Here, unilateral BDNF-LTP induction was associated with a bilateral increase in DG BrdU+ cell numbers (Fig. 5a–c). Blocking of the bilateral cell proliferation by unilateral ArcAS infusion, in turn, coupled this effect to unilateral BDNF-LTP induction. In previous work we have shown that the infused BDNF is restricted to the ipsilateral hippocampus and does not spread to the contralateral side^24^. LTP of perforant path-evoked transmission is itself a unilateral response coupled to NMDA receptor activation, BDNF-TrkB signaling and gene expression in the ipsilateral DG. LTP induction can have secondary effects in the network however, including bilateral changes in specific genes’ expression^33^. Inter-hemispheric communication in response to unilateral LTP has been demonstrated in fMRI studies in rats and appears to be mediated by activation of the mossy cell-commissural pathway to the contralateral DG^34^,^35^. Enhanced mossy cell-commissural transmission has in turn been suggested to function in the bilateral coordination of the DG networks involved in pattern separation and sequence learning^36^. While the current results link unilateral BDNF-LTP to bilateral proliferation, the implicated mechanisms are unknown and we do not exclude a potential role for direct effects of exogenous BDNF on NPCs.

In addition to increased bilateral BrdU+ cell numbers in the SGZ (Fig. 5a), unilateral BDNF infusion was accompanied by a corresponding bilateral reduction of IdU+ /BrdU− cells in the hilus, SGZ and GCL (Fig. 5d). This suggests BDNF not only promotes the *de novo* proliferation of neuroprogenitor cells (IdU-/BrdU+cells), but also stimulates the re-proliferation of NPCs which divided less than 20 hours prior to BDNF infusion (IdU+/BrdU− cells). This new proliferative event reduced the IdU+/BrdU− cell population since proliferating cells that incorporated IdU (administered approximately 20 hours prior to BrdU) divided again in response to BDNF and incorporated BrdU, thus becoming double positive for both thymidine analogues (IdU+/BrdU+cells) (Fig. 1). Due to the impossibility of distinguishing single- (IdU−/BrdU+; cells that divided once in response to BDNF) and double-labeled cells (IdU+/BrdU+; cells that proliferated twice in the 24 hours prior to sacrifice), both cell populations were labeled as BrdU+ cells. This categorization thus explains why BrdU+ cells increased in response to BDNF (Fig. 5a) while IdU+ /BrdU− cells declined (Fig. 5d).

Previously we reported Arc/Arg3.1 expression in BrdU+ cells from a very early post-mitotic age - as early as 1 day after cell division^10^. In the present study, detailed confocal analysis revealed that all cells proliferating in response to BDNF infusion expressed Arc/Arg3.1 (BrdU+/Arc+cells) (Fig. 6a). No changes were found in the total number of BrdU+/Arc− cells (Fig. 6d). Under basal conditions, only 1–5% of the total DG BrdU+cell population is double-labelled with Arc/Arg3.1. Remarkably, the percentage of Arc/Arg3.1-expressing BrdU+cells rose to more than 30% after BDNF infusion (Fig. 6a). It is important to emphasize that this bilateral increase in BrdU+/Arc+ cells was blocked by unilateral ArcAS infusion. We thus consider it likely that the bilateral effects associated with BDNF infusion are a consequence of unilateral BDNF-LTP induction. Moreover, there appear to be two distinct populations of BrdU+NPCs, one that proliferates and expresses Arc/Arg3.1 in response to BDNF-LTP induction (BrdU+/Arc+cells) and one that is insensitive to LTP induction (BrdU+/Arc− cells).

The mechanism underlying the bilateral increase in Arc/Arg3.1 expression in a subpopulation of young NPCs is presently unknown. Previously, we examined effects of unilateral high frequency stimulation-induced LTP (HFS-LTP) on Arc/Arg3.1 expression in newborn neurons^10^ and were surprised to find Arc/Arg3.1 labelling in early post-mitotic cells. Moreover no differences were found between the HFS-treated and contralateral DG in percentage of BrdU+/Arc+cells. Although we, then suggested that the lack of inter-hemispheric difference in BrdU+/Arc+ cell numbers in response to HFS-LTP was caused by the refractory nature of newborn cells to synaptically-evoked Arc/Arg3.1 expression, we now hypothesize that both HFS-LTP and BDNF-LTP promote bilateral pro-neurogenic effects and Arc/Arg3.1 expression in newborn cells through a similar mechanism. Since, it has been shown that the percentage of hippocampal BrdU+ cells expressing Arc/Arg3.1 increases with their age^10^, BrdU+/Arc+ cells might be generated by the division of a subgroup of older NPCs which are quiescent under basal conditions but capable of undergoing rapid proliferation in response to LTP induction at perforant path-granule cell synapses. Furthermore, since Arc/Arg3.1 expression in newborn cells is associated with their long-term survival and neuronal differentiation^10^, this would provide a mechanism by which LTP induction directs neurogenesis towards the generation of new neurons (in contrast to glial cells).

The present findings suggest a role for Arc/Arg3.1 in the mechanism underlying BDNF-mediated pro-neurogenic actions, possibly within the intracellular machinery which controls induction and/or maintenance of LTP. To further examine Arc/Arg3.1’s role in hippocampal neurogenesis, we investigated NPC proliferation and newborn cell survival in Arc/Arg3.1-knockout mice. While these transgenic mice were generated almost a decade ago^18^, no studies to date have explored the consequences of Arc/Arg3.1 deficiency on hippocampal neurogenesis. We hypothesized that if Arc/Arg3.1 regulates basal rates of NPC proliferation, a general reduction in BrdU+ cells would be clearly detectable in Arc/Arg3.1-deficient mice compared to wild-types. Basal proliferation rates may be unaffected however if Arc/Arg3.1 works selectively in activity-dependent regulation of neurogenesis, such as in response to LTP induction. Our results appear to support the latter as no changes were found in the total number of DG BrdU+ cells in ArcArg3.1-deficient mice sacrificed after 24 hours or 28 days compared to wild-type controls (Fig. 8e). Although our data suggest a secondary involvement of Arc/Arg3.1 in neurogenesis possibly related to its role in LTP generation and maintenance, we acknowledge these findings were obtained using homozygous Arc/Arg3.1-knockout mice whereas previous studies using other knockout lines have shown that functional loss of a gene due to a null mutation can often be compensated for, e.g. by their paralog(s). Granted we do not have the data to either confirm or reject this possibility in our Arc/Arg3.1-deficient mouse model, it will be important to explore the role of Arc gene/protein in the regulation of basal hippocampal neurogenesis using a conditional Arc/Arg3.1-knockout mouse model.

Our current findings point toward LTP induction as a primary mechanism underlying BDNF-mediated pro-neurogenic actions. Moreover, they illustrate an important yet complex role for the immediate-early gene, Arc/Arg3.1 and its protein in the BDNF-LTP-neurogenesis interplay. We propose the following scenario: **1**) BDNF-induced expression of Arc/Arg3.1 in mature DG cells is required for BDNF-LTP^17^; **2**) BDNF-LTP activates mechanisms/signaling which lead to rapid bilateral proliferation of NPCs and **3**) Arc/Arg3.1 expression characterizes the progeny of a subpopulation of quiescent NPCs which proliferate in response to BDNF-LTP. Taken together, these findings could hold relevance for learning and memory processes given the central role of BDNF in their regulation^4^,^37^,^38^,^39^. In the DG, acute BDNF signaling is implicated in pattern separation, a process by which similar experiences or events are transformed into discrete, non-overlapping memory representations, particularly during the consolidation of pattern-separated memories^40^. Acute local BDNF infusion facilitates this consolidation, a process requiring adult-born neurons^41^, although it is not yet known whether BDNF acts on mature or immature neurons. Notably, impaired pattern separation has been linked to increased anxiety. Failed pattern separation, perhaps consequent to impaired BDNF response/signaling and/or dysfunctional hippocampal neurogenesis, may result in the generalization of previously encountered aversive events to new “innocuous” experiences as seen in individuals with panic and post-traumatic stress disorder^42^. Remarkably, BDNF administration, both intra-hippocampal and peripheral, has been associated with antidepressant effects in behavioral models of anxiety and depression^12^,^13^,^43^, supporting the hypothesis that altered BDNF signaling and aberrant regulation of neurogenesis are central in the pathophysiology of these disorders. Our results add a new piece to the complex puzzle of BDNF signaling and point towards Arc/Arg3.1 as a possible intracellular mediator underlying BDNF-mediated stimulation of NPCs/immature DG neurons. Although this protein is commonly known as a direct modulator of glutamatergic synapses, recent work has shown that Arc/Arg3.1 also enters the nucleus and regulates gene expression^44^. It is tempting to speculate that Arc/Arg3.1 function in newborn immature cells, prior to synapse formation, involves such a nuclear mechanism. Further studies are needed however to define the relevance and specificity of Arc/Arg3.1 in the transduction of BDNF-mediated signals with particular emphasis on its involvement in the regulation of NPC proliferation and integration of newborn cells in the adult hippocampal circuitry.

## Materials and Methods

### Animals

Four-month old Male Sprague Dawley rats (Møllegårds Avlslaboratorium, Denmark; *n* = 33, 250–350 g), four-month old male transgenic *Arc/Arg*3.1-deficient mice (*n* = 12, 20 gr) and wild-type controls (*n* = 12, 20 gr) were used. Animals were individually housed with *ad libitum* access to food and water under climate-controlled conditions (22º ± 1 °C). They were maintained on a 12-hour light/dark cycle (light on at 07:00) and received at least 7 days to acclimatize to their new environmental conditions prior to the experiments. This investigation was designed to minimize the number of animals and suffering, and carried out in accordance with the Norwegian and German Regulation on Animal Experimentation, and the European Convention for the Protection of Vertebrate Animals used for scientific purposes. All experimental procedures were reviewed and approved by the local animal welfare body and performed according to University of Bergen Guidelines for the Care and Use of Laboratory Animals (project id:4307).

Homozygous Arc/Arg3.1-deficient mice were provided by Prof. D. Kuhl and generated as previously described^18^. Briefly, genomic fragments of *Arc/Arg3*.1 were isolated from a λ phage genomic library (AB-1) prepared from 129/Sv(ev) embryonic stem (ES) cells. A 4 kb fragment encompassing the promoter and 5′UTR was subcloned into pBLUESCRIPT (Stratagene), and a 3.7 kb fragment covering the whole open reading frame (ORF) and 3′UTR was subcloned into pZErO-1. These plasmids were used for generation of a targeting construct. For additional information on targeting, genotyping, and anatomical analysis readers are referred to Plath *et al*.^18^.

### Experimental design

In this study, the effects of intra-dentate unilateral BDNF infusion on hippocampal NPC proliferation were examined in male rats. The role of Arc/Arg3.1 protein on BDNF-mediated proneurogenic actions was also investigated by locally infusing ArcAS prior to BDNF administration (Fig. 1). Finally, the effects associated with reduced Arc/Arg3.1 expression on adult hippocampal neurogenesis were explored using adult Arc/Arg3.1-deficient mice.

Rats were randomly assigned to 4 experimental groups:

1. Cytochrome C (n = 9): received a local unilateral infusion of cytochrome C in the DG;
2. BDNF (n = 8): received a local unilateral infusion of BDNF in the DG;
3. BDNF+ArcAS (n = 8): received a local unilateral infusion of Arc/Arg3.1 antisense oligodeoxynucleotides in the DG prior to BDNF administration;
4. BDNF+ArcASScr (n = 8): received a local unilateral infusion of scrambled Arc antisense oligodeoxynucleotides in the DG prior to BDNF administration.

Arc/Arg3.1-deficient (TG) and wild-type (WT) mice were divided across 4 groups to investigate effects associated with reduced Arc/Arg3.1 expression on cell proliferation and survival by administering BrdU respectively, 24 hours or 28 days prior to sacrifice:

1. TG proliferation: Arc/Arg3.1-deficient mice (n = 6) sacrificed 24 hours after BrdU;
2. WT proliferation: wild-type mice (n = 6) sacrificed 24 hours after BrdU;
3. TG survival: Arc/Arg3.1-deficient mice (n = 6) sacrificed 28 days after BrdU;
4. WT survival: wild-type mice (n = 6) sacrificed 28 days after BrdU.

### BrdU and IdU Injections

To compare NPC proliferation changes before and after BDNF and/or ArcAS infusion, rats were injected with thymidine analogues 5-iodo-2′-deoxyuridine (IdU: 24 hours before sacrifice) and 5-bromo-2′-deoxyuridine (BrdU: 4 hours prior to sacrifice). Both IdU (230 mg/kg, 100 mg/ml; Sigma-Aldrich) and BrdU (200 mg/kg, 100 mg/ml; Sigma-Aldrich) were administered intraperitoneally (i.p.) in single injections (Fig. 1). IdU and BrdU were dissolved in 0.9% NaCl solution with an equal molarity of 0.65 M and a pH ranging from 8 to 8.5.

Mice received only BrdU, through a single i.p. injection (100 mg/kg; 50 mg/ml) administered 24 hours (proliferation) or 28 days (survival) prior to sacrifice.

### Intra-dentate BDNF infusion and electrophysiology

**Figure 2:**
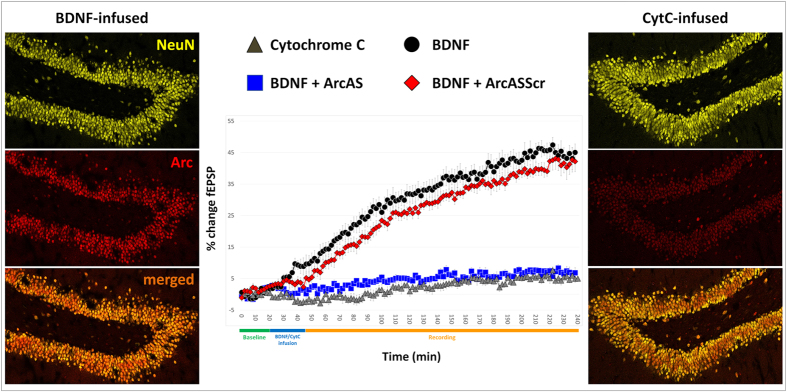
Time-course plot illustrating changes in perforant path-DG evoked fEPSP slope expressed in percentage of baseline. Values are means ± SEM. CytC, BDNF, BDNF+ArcAS and BDNF+Arc ASScr infusion points are indicated. Average field potential traces (10 sweeps) collected at the end of baseline and during the recording (post-BDNF) phases are shown. Calibration: 5 mV, 2 ms. (n = 8/9 per group). Representative photomicrographs illustrating NeuN and Arc/Arg3.1 expression changes in the DG following unilateral BDNF infusion (BDNF-infused) or unilateral CytC infusion (CytC-infused).

BDNF was locally and unilaterally infused in the DG as previously described^17^,^24^,^26^. Briefly, rats were anesthetized with urethane (1.4–1.8 g/kg i.p.) and BDNF was infused immediately above the dorsal DG, in deep stratum-lacunosum-moleculare of field CA1, approximately 300 μm above the nearest medial perforant path-granule synapses and 700 μm above the hilar recording site. Infusion solutions of BDNF (2 μg/2 μl; Alomone Labs., Jerusalem) and cytochrome C obtained from yeast (Sigma, St Louis, MO, USA) were dissolved in sodium phosphate buffer, pH 7.0 and delivered by infusion pump at a rate of 80 nl/min over 25 minutes. With a molecular weight and charge similar to BDNF, cytochrome C was infused as a protein control as it has no effect on basal synaptic transmission or several signal transduction pathways that have been studied^25^,^26^,^45^,^46^. This administration protocol resulted in an immediate and potent LTP induction (Fig. 2). Signals from the dentate hilus were amplified, filtered (1 Hz to 10 kHz) and digitized (25 kHz). Acquisition and analysis of field potentials was accomplished using DataWave Technologies WorkBench software (Longmont, CO, USA). Responses were normalized to baseline and statistics were based on average values obtained during the baseline (from minute −20 to 0), infusion (from 0 to minute 25) and final 20 min period of recording (from minute 170 to 195) (Figs 1 and 2).

### Oligodeoxynucleotides (ODNs): Arc anti-sense and Arc scrambled ODNs

Chimeric ODNs containing phosphorothioate linkages between the three bases on the 5′ and 3′ ends and phosphodiester internal linkages were synthesized, HPLC purified, ultrafiltrated, and sterilized (BIOGNOSTIK® GmbH, Göttingen, Germany). The main Arc/Arg3.1 antisense ODN (ArcAS) used was directed against a 20-mer sequence (bases 209–228) covering the Arc start site. The ArcAS ODN sequence used in our experiment was 5′GTC CAG CTC CAT CTG CTC GC 3′while the scrambled Arc ODN sequence was 5′CCT GCT GAC CTC CGT ATG CC 3′. Scrambled Arc ODN sequence (ArcASScr), containing the same base composition of ArcAS sequence in a randomized order, served as control. ODNs did not contain motifs such as G-quartets, kinase domains or zinc fingers, and search of the European Molecular Biology Laboratory databases revealed no potential off-target genes (with significant homology and open secondary structure). Arc antisense and scrambled ODNs were infused at a rate of 80 nl/min over 12.5 min (Fig. 1). Although in this study the effects of anti-sense ODNs on Arc translation was not directly demonstrated, these effects have been extensively investigated in three previous studies. Initially, we showed that BDNF-LTP was accompanied by the selective upregulation of Arc mRNA and protein in the dentate gyrus^26^. In a following study, the effect of actinomycin D on Arc upregulation was assessed^24^. This transcription inhibitor blocked the upregulation of Arc protein in the dentate gyrus and induction of BDNF-LTP, strengthening the link between Arc and BDNF-LTP. Arc protein levels were measured by Western blot analysis of homogenates obtained from micro-dissected dentate gyrus, CA1 and CA3 regions. In the final study, the effects of Arc anti-sense ODN infusion on Arc protein expression (by quantitative immunoblot analysis) and high-frequency stimulation-induced LTP were investigated^17^. Our data revealed that Arc protein expression was reduced to 55 ± 10% in response to Arc AS administration compared to ArcAS scrambled-treated controls^17^ confirming the efficacy and specificity of ArcAS ODNs in blocking Arc translation and suggesting a critical role for Arc protein and its synthesis for LTP consolidation.

### Tissue collection

Rats were sacrificed after 195 minutes of recording (Fig. 1) and transcardially perfused with 4% paraformaldehyde in 0.1 M sodium phosphate buffer (pH 7.4). Mice were perfused with the same fixative 24 hours or 28 days after receiving BrdU. Brains were removed and post-fixed in the same solution for 24 hours at 4 °C, before being transferred to sodium phosphate buffer (NaPB 0.02 M, pH 7.4) and stored at 4 °C. Following cryoprotection of the brains by overnight immersion in a 30% sucrose solution, 35 μm coronal serial sections were prepared on a cryostat microtome. Sections were collected in NaPB with sodium azide and stored at 4 °C. Immunohistochemical stainings were performed using subsets of coronal sections.

### Immunohistochemistry

Every sixth section throughout the rostral/caudal extent of the hippocampus (Bregma −2 to −6 in rats and −1.2 to −2.6 in mice) was collected and coded before immunohistochemical processing and analysis to ensure objectivity. All stainings were performed on free-floating sections under continuous agitation.

#### Arc/Arg3.1 expression

BDNF infusion results in robust LTP induction associated with a strong increase of Arc/Arg3.1 expression in the dentate gyrus. Immunofluorescent Arc/Arg3.1 labeling was thus performed to evaluate the effect/success of BDNF-mediated LTP induction (Fig. 2). Sections were incubated in the primary polyclonal rabbit anti-Arc/Arg3.1 antibody raised against amino acids 1–300 of the Arc protein (H300; SantaCruz Biotechnology, Santa Cruz CA, sc-15325; 1:500 dilution in NaPBS 0.02 M, pH7.4) for 72 hours at 4 °C. Subsequently, sections were washed with NaPBS and incubated at room temperature with Alexa-647 donkey anti-rabbit antibody (Molecular Probes, A-31573) (Fig. 2). Specificity of results obtained with the H300 antibody, was confirmed by comparative staining performed with an alternative anti-Arc/Arg3.1 monoclonal mouse antibody raised against amino acids 264–385 (BD Biosciences; San Jose, CA; #612603).

#### IdU and BrdU immunohistochemistry

**Figure 3:**
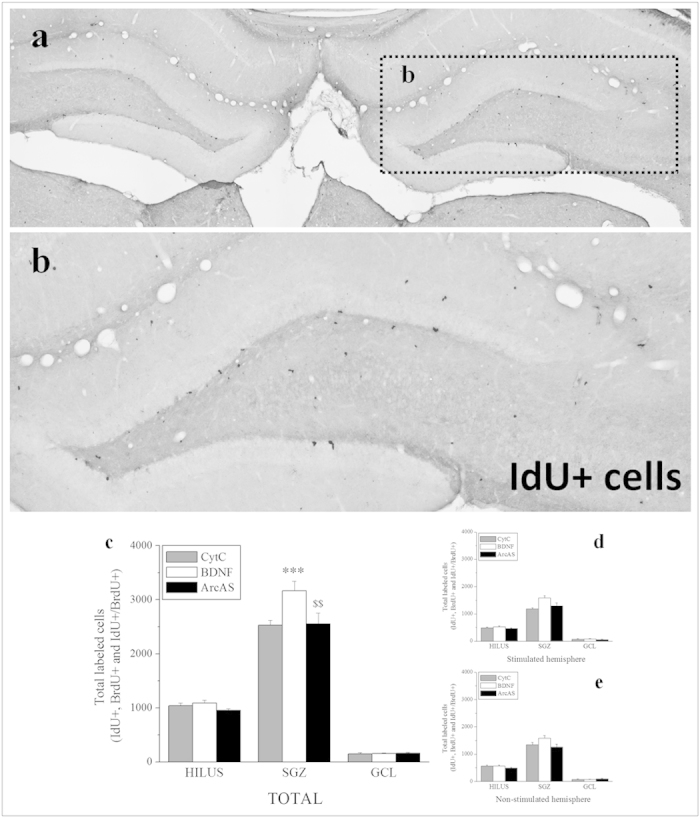
Total IdU+ and BrdU+ cells. Representative photomicrographs illustrating IdU immunohistochemistry in the dentate gyrus following BDNF infusion (**a**, 4× magnification; **b**, 10× magnification). Since the monoclonal anti-IdU antibody used here could not discriminate between IdU or BrdU, it labeled all dividing IdU+ and/or BrdU+ cells. Total numbers of IdU-labeled cells thus represent a combination of three distinct cell populations: IdU+/BrdU−, IdU−/BrdU+ and IdU+/BrdU+ cells. Graph illustrating average total numbers of total labeled cells in the hippocampal hilus, subgranular zone (SGZ) and granule cell layer (GCL) per group (n = 8/9) ± SEM (**c**). Graphs illustrating average numbers of labeled cells in the stimulated (BDNF-infused, **d**) and contralateral hemisphere (**e**). The * and $ symbols represent significant effects compared to CytC- or BDNF-treated animals, respectively. One, two or three symbols represent p < 0.05, p < 0.01, p < 0.001, respectively.

Peroxidase labeling of IdU+ and BrdU+ cells was performed as previously described^10^,^47^. Labeling included the following pretreatment steps: preincubation in 0.3% H_2_O_2_ for 15 minutes to reduce endogenous peroxidase activity, DNA denaturation (50% formamide/2 × SSC, pH7.0, 65 °C, 120 min) and acidification (2N HCl, 30 min). The primary antibodies, monoclonal mouse anti-IdU (BD Biosciences 347580; 1:800) and monoclonal rat anti-BrdU (Oxford Biotechnology, OBT0030CX; 1:800), were diluted in NaPBS 0.02 M, pH7.4. The secondary antibodies, biotin-SP-conjugated horse-anti-mouse (Vector ABC Elite kit, Vector Laboratories, Burlingame, CA, USA; 1:400) and donkey-anti-rat (Jackson ImmunoResearch Laboratories, Inc., West Grove, PA, USA; 1:400) were diluted in NaPBS 0.02 M, pH7.4). Sections were subsequently incubated in ABC solution (Vector ABC elite kit, Vector Laboratories, Burlingame, CA, USA) and rinsed in NaPBS. Reaction products were visualized by adding diaminobenzidine as chromogen and 1% H_2_O_2_ for 30 min. Labeled sections were washed, mounted on slides, dehydrated and coverslipped with DPX (Fig. 3a,b).

#### Immunofluorescent IdU/BrdU/Arc triple-labeling

**Figure 4:**
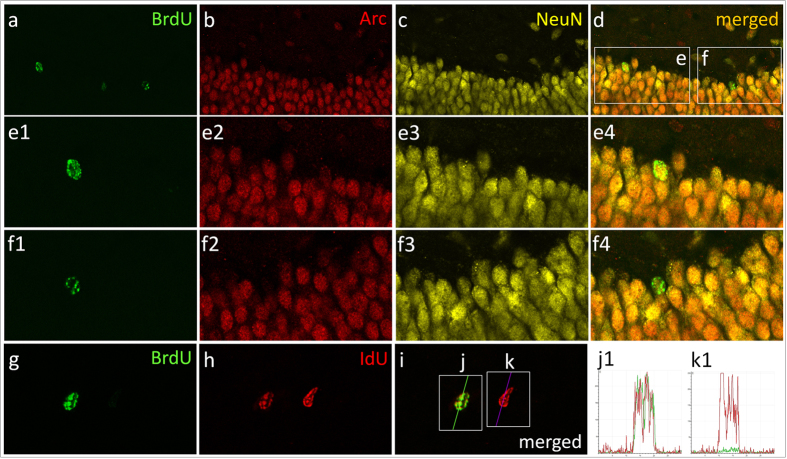
Representative confocal photomicrographs depicting triple immunofluorescent BrdU/Arc/NeuN and double BrdU/IdU labeling from an animal receiving BDNF infusion. Photomicrographs illustrating both BrdU+/Arc+/NeuN+and BrdU+/Arc−/NeUN+ cells (**a–d**). High magnification images of inserts (**e**), illustrating a BrdU+/Arc+/NeuN+ cell (**e**_**1**_**–e**_**4**_,**f**) showing a BrdU+/NeUN+ cell negative for Arc (**f**_**1**_**–f**_**4**_). The latter images demonstrate that although Arc seems ubiquitously expressed in the DG, some IdU+ and/or BrdU+ cells were clearly Arc negative. Representative confocal photomicrographs depicting two different proliferating cells positive for BrdU and/or IdU (**g–k**): the first cell is clearly double positive for IdU and BrdU (**j,j**_**1**_) while the second is only positive for IdU (**k,k**_**1**_).

**Figure 5:**
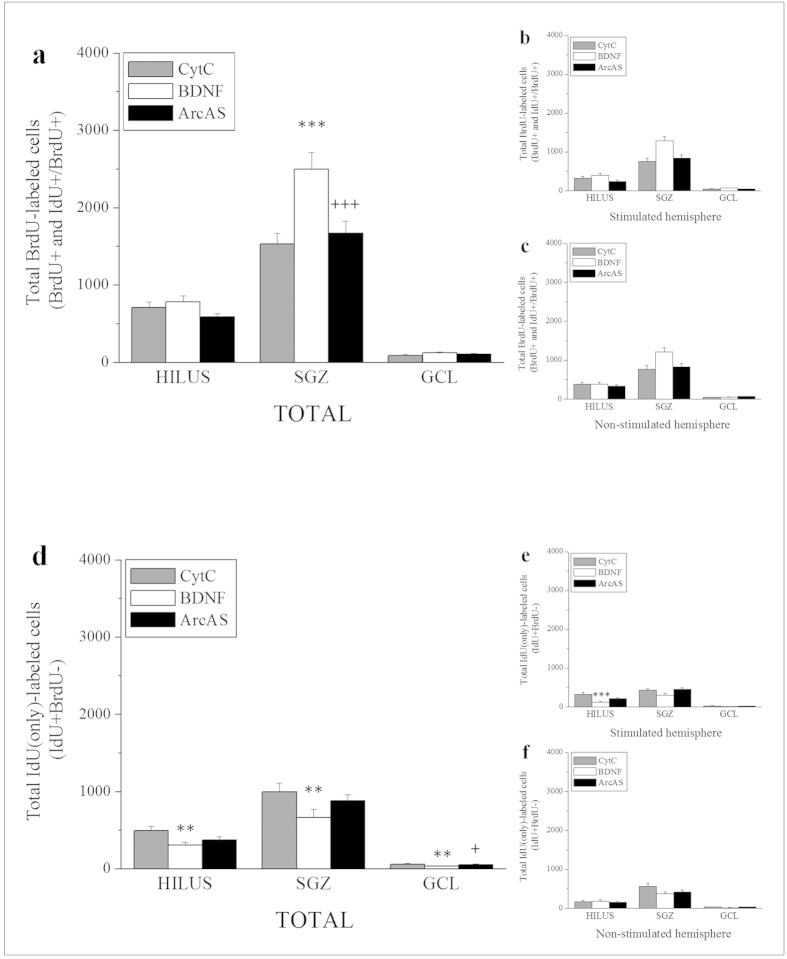
Graphs illustrating average total numbers of BrdU+(IdU−/BrdU+ and IdU+/BrdU+) cells in the hippocampal hilus, subgranular zone (SGZ) and granule cell layer (GCL) per group (n = 8/9) ± SEM (**a**) as well as average numbers of BrdU+ cells in the stimulated (BDNF-infused, **b**) and contralateral hemisphere (**c**). Graphs illustrating averages of total numbers of IdU(only)+ or IdU+/BrdU- cells in the hippocampal hilus, subgranular zone (SGZ) and granule cell layer (GCL) per group (n = 8/9) ± SEM (**d**) as well as average numbers of IdU(only)+ cells in the stimulated (BDNF-infused, **e**) and contralateral hemisphere (**f**). The * and + symbols represent significant effects compared to CytC- or BDNF-treated animals, respectively. One, two or three symbols represent p < 0.05, p < 0.01, p < 0.001, respectively.

**Figure 6:**
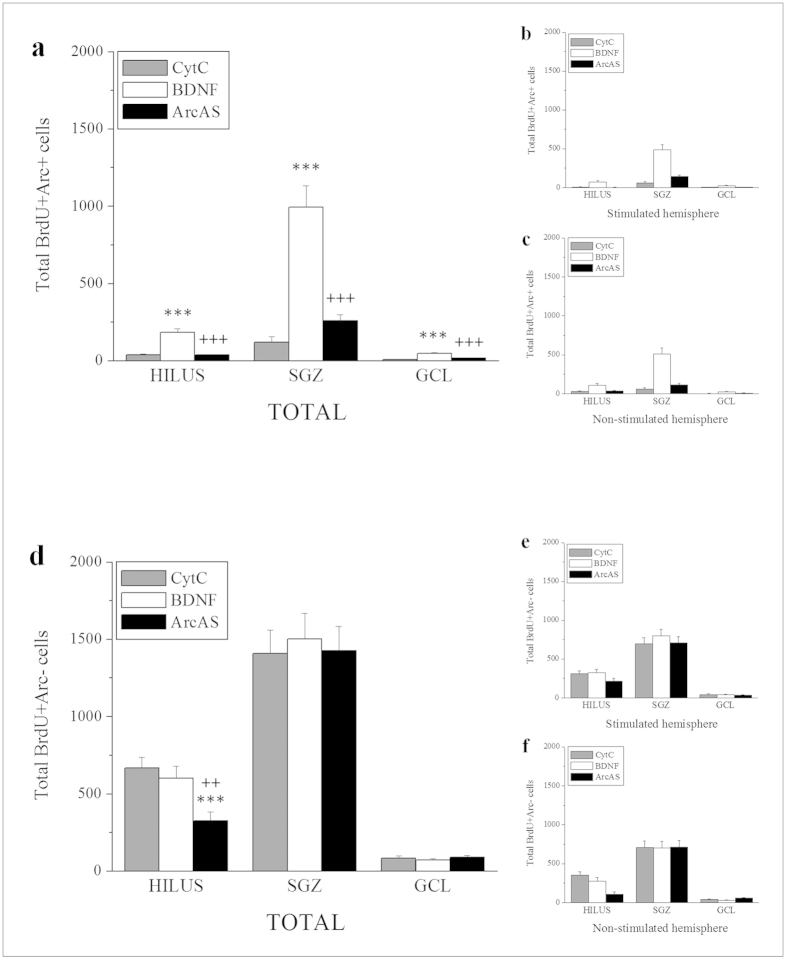
Graphs illustrating average total numbers of IdU−/BrdU+/Arc+ (IdU−/BrdU+/Arc+and IdU+/BrdU+/Arc+) cells in the hippocampal hilus, subgranular zone (SGZ) and granule cell layer (GCL) per group (n = 8/9) ± SEM (**a**) as well as average numbers of BrdU+/Arc+ cells in the stimulated (BDNF-infused, **b**) and contralateral hemisphere (**c**). Graphs illustrating average total numbers of BrdU+/Arc− (IdU−/BrdU + /Arc− and IdU+/BrdU+/Arc−) cells in the hippocampal hilus, subgranular zone (SGZ) and granule cell layer (GCL) per group (n = 8/9) ± SEM (**d**) as well as average numbers of BrdU+/Arc− cells in the stimulated (BDNF-infused, **e**) and contralateral hemisphere (**f**). The * and + symbols represent significant effects compared to CytC− or BDNF-treated animals, respectively. One, two or three symbols represent p < 0.05, p < 0.01, p < 0.001, respectively.

**Figure 7:**
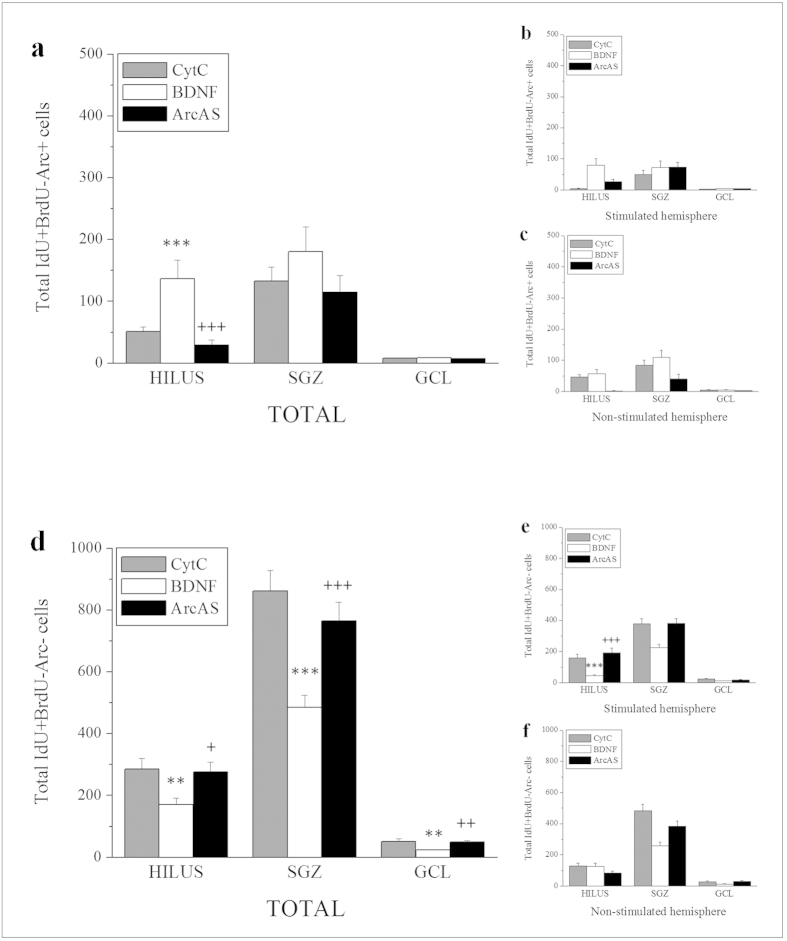
Graphs illustrating average total numbers of IdU+/BrdU−/Arc+ cells in the hippocampal hilus, subgranular zone (SGZ) and granule cell layer (GCL) per group (n = 8/9) ± SEM (**a**) as well as average numbers of IdU+/BrdU−/Arc+ cells in the stimulated (BDNF-infused, **b**) and contralateral hemisphere (**c**). Graphs illustrating average total numbers of IdU+/BrdU-/Arc- cells in the hippocampal hilus, subgranular zone (SGZ) and granule cell layer (GCL) per group (n = 8/9) ± SEM (**d**) as well as average numbers of IdU+/BrdU-/Arc- cells in the stimulated (BDNF-infused, **e**) and contralateral hemisphere (**f**). The * and + symbols represent significant effects compared to CytC- or BDNF-treated animals, respectively. One, two or three symbols represent p < 0.05, p < 0.01, p < 0.001, respectively.

**Figure 8:**
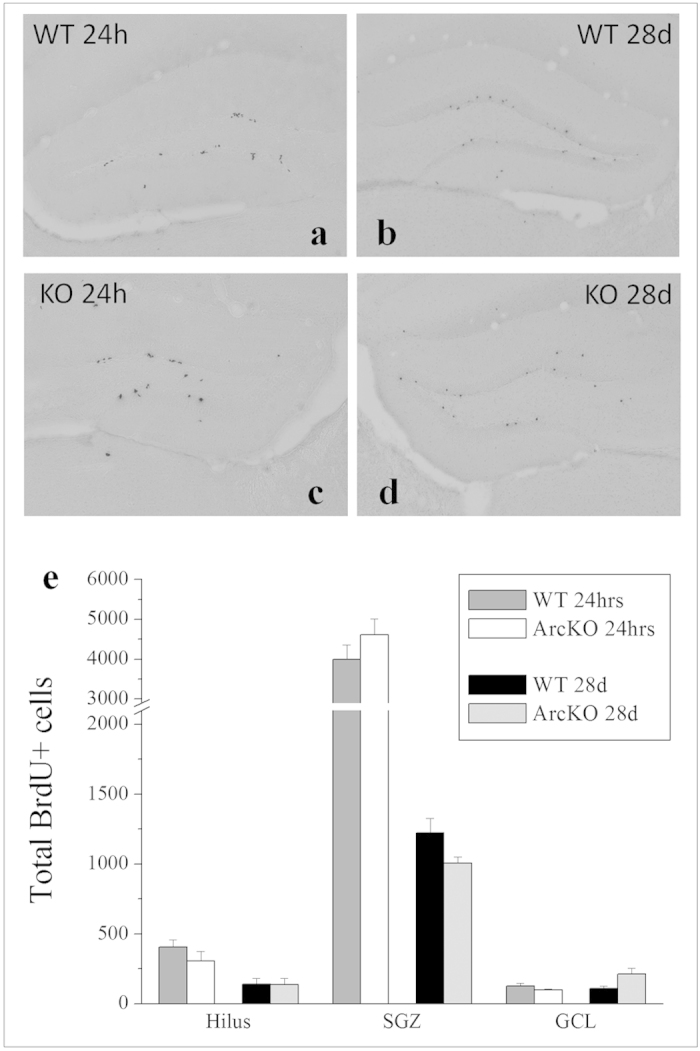
Total BrdU-labeled cells in Arc/Arg3.1-deficient and wild-type mice. Representative photomicrographs illustrating BrdU immunohistochemistry in the DG of wild-type and Arc/Arg3.1-deficient mice 24 hours and 28 days following BrdU administration (**8a–d**). Graph illustrating average total numbers of BrdU-labeled cells ± SEM in the hippocampal hilus, subgranular zone (SGZ) and granule cell layer (GCL) of wild-type mice and Arc/Arg3.1-deficient mice sacrificed 24 hours and 28 days after BrdU administration, respectively (n = 6) (**8e**).

For multi-labeling, every 12th section per animal was denaturated (50% formamide/2 × SSC, pH7.0, 65 °C, 120 min and 2N HCl, 30 min) and incubated overnight in monoclonal rat anti-BrdU (Oxford Biotechnology, OBT0030CX; 1:300), monoclonal mouse anti-IdU (BD Biosciences 347580; 1:800) and polyclonal rabbit anti-Arc/Arg3.1 H300 (SantaCruz Biotechnology, Santa Cruz CA, sc-15325; 1:100) diluted in NaPBS 0.02 M, pH7.4. Sections were subsequently incubated in Alexa-488 donkey anti-rat (Molecular Probes; A21208; 1:200) followed by Alexa-647 donkey anti-mouse (Molecular Probes; A31571; 1:200) and Alexa-555 donkey anti-rabbit (Molecular Probes; A31572; 1:200) antibodies. Finally, sections were mounted on slides and cover-slipped using Vectashield mounting medium (Vector Laboratories, Burlingame, CA, USA) (Fig. 4).

### Image Analysis and Quantification

Quantification of IdU+ cells in rats and BrdU+ cells in mice was conducted using a modified unbiased stereology protocol^10^,^48^. All labeled cells in the granule cell layer (GCL), subgranular zone (SGZ) and hilus were counted (at 200×) regardless of size or shape using a Nikon Eclipse 80i microscope (Tokyo, Japan) coupled to a Nikon DS-5M camera. Cells were considered as being in the subgranular zone if they were located in or adjacent to the SGZ. Cells located more than two cell widths away from the SGZ were considered hilar. To facilitate counting, cell clusters were examined at 400×. Quantification was conducted bilaterally and the total number of labeled cells was estimated by multiplying the number of cells counted in every sixth section by 6 and reported as the total numbers of labeled cells (mean and SE). As rats received both IdU and BrdU (Fig. 1), it should be noted that the IdU antibody from BD Biosciences recognizes both analogs, and so will label cells which have incorporated IdU and/or BrdU. This means that the total number of labeled cells in rats thus represents a combination of three distinct cell populations: IdU(only)+ cells, BrdU(only)+ and double IdU+/BrdU+ cells.

For multi-labeling, percentages of IdU+ and/or BrdU+ cells were determined by analyzing, in each animal, 50–100 randomly selected labeled cells throughout the GCL and SGZ using a Leica TCS SP2 AOBS confocal microscope (Leica Microsystems, Heidelberg GmbH). Care was taken to verify double-labeling and to control for false positives by examining double-positive nuclei in their *z*-axis and rotating them in orthogonal x-y planes using a 40× objective (1 μm steps). To exclude potential cross-bleeding between detection channels, double-labeling was imaged in sequential scanning mode.

Given the distinct specificity profiles of the two antibodies (IdU antibody’s dual specificity for IdU/BrdU and the BrdU antibody’s sole specificity for BrdU), we applied a triple-labeling protocol using species-specific secondary antibodies conjugated to different fluorophores to identify IdU- and/or BrdU-labeled cells. Following this protocol, BrdU+ cells were labeled by both green (488-conjugated) and red (647-conjugated) fluorescent secondary antibodies, whereas IdU(only)+ cells which did not proliferate a second time in response to BDNF infusion were labeled by the red fluorescent antibody only (Fig. 4). If an IdU(only)+ cell divided again in response to BDNF, it would also incorporate BrdU thus becoming double labeled (IdU+/BrdU+). An important limitation of this protocol lies in the inability to discriminate between IdU+ cells which re-divided in response to BDNF and incorporated BrdU (double IdU+/BrdU+ cells) and IdU-negative neuroprogenitor cells which proliferated only once in response to BDNF (BrdU(only)+). Since the BD IdU antibody cannot distinguish between IdU and BrdU, all BrdU-labeled cells (BrdU(only)+ and double IdU+/BrdU+) will be both green and red. Finally, depending upon Arc/Arg3.1 expression (yellow; 555-conjugated antibody), cells which divided within the timeframe of our experiment (Fig. 1) could appear either double (IdU(only)+/Arc+) or triple-positive (IdU+/BrdU+/Arc+) as illustrated in Fig. 4.

### Statistics

SPSS was used to perform the analysis of electrophysiological and immunohistochemical data. General linear model for repeated measures was used to analyze group effects of electrophysiological results. Within-Subjects variable was time (time1 = average of last 10 minutes of baseline; time2 = average of first 10 minutes of CytC/BDNF infusion; time3 = average of final 10 minutes of recording) while Between-Subject factor was treatment (CytC vs BDNF vs BDNF+ArcAS vs BDNF+ArcASScr). Two-way ANOVA was instead used to analyze immunohistochemical results. Independent variables included treatment (CytC vs. BDNF vs. ArcAS) and hemisphere of infusion (stimulated vs. non-stimulated). Results were considered significant when p ≤ 0.05. Pairwise multiple comparisons (Sidak post-hoc methods) were applied to more accurately assess the source of variation between groups.

## Additional Information

**How to cite this article**: Kuipers, S. D. *et al*. BDNF-induced LTP is associated with rapid Arc/Arg3.1-dependent enhancement in adult hippocampal neurogenesis. *Sci. Rep.* **6**, 21222; doi: 10.1038/srep21222 (2016).
